# Apelin and Atrial Fibrillation: The Role in the Arrhythmia Recurrence Prognosis

**DOI:** 10.1155/2018/5285392

**Published:** 2018-03-12

**Authors:** Agata Salska, Michał Dziuba, Witold Salski, Krzysztof Chizynski, Marzenna Zielinska

**Affiliations:** ^1^Intensive Cardiac Therapy Clinic, Medical University, Lodz, Poland; ^2^Department of Occupational Diseases and Environmental Health, Nofer Institute of Occupational Medicine, Lodz, Poland

## Abstract

Apelin is a novel peptide of wide expression and multiple biological functions including the crucial role in cardiovascular homeostasis. The apelin role in the pathophysiology of heart rhythm disorders is considered, although the reports are scarce so far. The purpose of this study is to investigate the potential utility of apelin as a marker of arrhythmia recurrence after direct-current cardioversion (DC). The prospective, observational study included 60 patients (aged 41–86; 30% female) with nonvalvular, persistent atrial fibrillation from the group of 204 consecutive patients scheduled for DC during the 12-month period (from May 2010 to May 2011) in the Cardiology Clinic Medical University of Lodz, Poland. The study group was divided into SCD (successful DC), 45 (75%) patients, and NDC (nonsuccessful DC), 15 (25%) patients. Within the SCD group, the subgroups were distinguished depending on the time sinus rhythm maintenance after DC: up to 7 days (SDC-7), 11 patients; 7 to 30 days (SDC-30), 12 patients; over 90 days (SDC-90), 22 patients. Patients were evaluated during the hospitalization and within the 3-month follow-up period. The apelin level was determined within the plasma samples collected at the admission, using the commercially available enzyme-linked immunosorbent assay (ELISA) Kit for apelin-36. It was found that the median value of initial apelin in the subset of patients from groups NDC + SDC-7 + SDC-30 is significantly higher than from group SDC-90 (*p* = 0.0463); there was no relationship between NDC and SCD overall. Neither of the compared subgroup pairs revealed statistically significant correlation between the proBNP concentration and the DC effectiveness in our population. In conclusion, in our study, proBNP was not a marker of arrhythmia recurrence whereas higher apelin concentration at the admission indicated patients in whom DC was not effective or they had an arrhythmia recurrence within a month-period observation.

## 1. Introduction

Atrial fibrillation (AF) is the most frequent sustained cardiac arrhythmia in adults, which affects over 1% of the general population and becomes an important cause of the mortality and life quality deterioration [1, 2]. It causes significant morbidity and premature mortality, with the risk of death doubled, regardless of the other factors [3].

The therapeutic strategy in atrial fibrillation should be planed individually and include thromboembolism prophylaxis and the rhythm restoration or control [1]. One of the available therapy methods is the electric cardioversion, which is an effective way to restore the sinus rhythm [4]. There is a substantial risk of arrhythmia recurrence after cardioversion. It is estimated as 10% per year in patients after the first diagnosed AF incident. It increases by 5% each year [1]. The most important AF-recurrence risk factors include age, AF duration before the cardioversion, the number of previous relapses, the left atrium enlargement or dysfunction, coronary artery disease, or the mitral valve defect [1].

The new markers including biochemical substances of a potential role in the atrial fibrillation risk stratification are investigated. The researches are intended to deal with the improvement of the AF-associated clinical risk and/or recurrence rate assessment. The usefulness of such substances as troponin, natriuretic peptides, the inflammation, renal function, and coagulation markers was proved [5]. It was proved that lone AF in patients with normal left ventricle ejection fraction is associated with elevated BNP/proBNP serum level; moreover, its level decreases within the sinus rhythm maintenance after DC and increases in case of arrhythmia recurrence. Some researcher however indicates that baseline BNP serum concentration is not associated with the risk of AF recurrence [6, 7].

Among other substances, the role of apelin in atrial fibrillation became an object of interest. Apelin is a novel peptide that was first isolated and named by Tatemoto et al. in 1998 [8]. It was named after the APJ endogenous ligand, as it was identified as the endogenous ligand for the previously recognised human APJ “orphan” receptor [8].

Apelin is encoded by a gene located on band q25-26.1 of the chromosome X and is synthesized as a pre-pro-apelin precursor peptide consisting of 77 amino acids, which is subsequently transformed into active isoforms consisting of 12 to 36 amino acids. Apelin applies its various regulating functions by acting on a transmembrane G protein-coupled APJ receptor, which indicates significant similarity to the angiotensin receptor (AT1) [9, 10].

Both apelin and APJ receptor are widely expressed in numerous cells and tissues of the human body including the central nervous system and peripheral tissues [9]. Apelin is also detectable in human plasma. Within cardiovascular system, it is present in the vascular smooth muscle cells, endocardial and endothelial cells of the heart chambers, and vascular endothelial cells of many vessels—coronary arteries, large conduit vessels, and small arteries and veins (e.g., kidney, lung, and adrenal gland vessels) [9]. It plays a role both in patophysiological and cardioprotective pathways. It has been proved that apelin has multiple functions within the cardiovascular homeostasis affecting the angiogenesis, vascular tone, and body fluid homeostasis or acting as a positive inotropic factor [8]. In human, it was shown that in early stages of the heart failure, plasma apelin level, parallel to BNP, rises and decreases with the progress of the disease [11].

As the apelin is produced and secreted in adipose tissue, it is also considered as an adipokine. As such, it may affect many of the metabolic pathways, especially in carbohydrate metabolism, where apelin/APJ system is interdependent with insulin. It also plays a role in the pathophysiology of the obesity, diabetes mellitus, and atherosclerosis [12].

Taking into account the discussed issue, it is important that apelin has also an influence on electrophysiological function of atrial myocytes, shortening the action potential, which was demonstrated in vitro on animal model [13].

Recently, in individual reports, it was shown the mean apelin level is significantly reduced in patients with isolated atrial fibrillation and normal heart in echocardiography, when compared to the healthy control [14], and it is also decreased in patients with other supraventricular tachyarrhythmias [15]. Moreover, in these patients, the maintenance of the sinus rhythm after cardioversion increases the apelin level [16]. Up to date, only one study evaluated the impact of the marker level for the risk of arrhythmia recurrence [17].

The aim of this study is to evaluate the potential impact of initial apelin-36 on the risk of AF recurrence after DC.

## 2. Materials and Methods

The prospective, observational study included 60 patients with nonvalvular persistent atrial fibrillation from the group of 204 consecutive patients scheduled for electric direct-current cardioversion (DC) during the 12-month period (from May 2010 to May 2011) in the Cardiology Clinic Medical University of Lodz, Poland. Patients were included after the exclusion of negative factors and signing an informed consent of the participation form. Patients were evaluated during hospitalization and at the 3-month follow-up period. The study was approved by the local Bioethics Committee.

The exclusion criteria were as follows:

- atrial flutter or atrial tachycardia; persistent atrial fibrillation with the duration of less than four weeks
- INR < 2.0 within the four weeks prior to DC
- electrolyte abnormalities; chronic kidney disease, defined as GFR < 60 ml/min/1.73 m^2^ or creatinine concentration > 1.5 mg/dl
- an acute or chronic inflammatory diseases; immunosuppressive therapy; antibiotics, steroids, or nonsteroid drugs intake
- active endocrine disorders; active cancer
- alcohol abuse
- a history of the rheumatic fever
- a history of the cardiac surgery ever or any surgery within the past 6 months
- state after the pacemaker or cardioverter-defibrillator implantation
- a history of the ineffective DC
- symptoms of heart failure in the ≥II hemodynamic scale according to the New York Heart Association (NYHA)
- data from echocardiography: intracardiac thrombus, left ventricle ejection fraction (LVEF) < 45%, the left atrium dimension > 55 mm measured in the parasternal long axis (LAX), left or right atrium longitudinal or transverse dimension > 70 mm measured in the four-chamber projection (dimension), valvular defects (moderate and severe aortic or mitral stenosis, valvular regurgitation > II degree).

In each patient, the detailed data on medical history were obtained. Patients underwent the physical examination, electrocardiogram (ECG), and transthoracic echocardiography. At the admission, the fasting laboratory tests including peripheral blood count, electrolytes, blood glucose, urea and creatinine, INR, and serum brain natriuretic propeptide (proBNP) were performed. In each case, an additional blood sample of 5.2 ml was collected, immediately centrifuged, and stored at temperatures below −70°C as recommended by the reagent manufacturer.

The apelin (isoform apelin-36) level was determined within the plasma samples using the commercially available enzyme-linked immunosorbent assay (ELISA) Kit for apelin-36 (Uscn Life Science Inc.). The assay employs a monoclonal antibody for human apelin-36 and is based on competitive inhibition enzyme immunoassay technique—detection range was 14.25–3000 pg/ml and intra- and interassay CVs were <10% and <12%, respectively.

Electric cardioversion was performed under the short-term general anesthesia after obtaining patients consent. The procedure was carried out subject to the confirmation of the AF wave in the cardiomonitor registration, using the transthoracic electrodes and the diphase energy of 200 J. If there was no sinus rhythm restoration, the DC was repeated with increasing energy of 300 and 360 J up to three repetitions.

DC was considered effective provided that the sinus rhythm was obtained during the first three electrical discharges. The heart rate was monitored on the screen of the cardiomonitor, and additionally, there was the ECG recording confirming the sinus rhythm required.

DC was considered ineffective if it could not restore the sinus rhythm after the three consecutive electrical discharges of increasing energy according to the scheme above. The ineffectiveness was confirmed by the presence of the AF wave in ECG.

Patients after successful DC were prospectively observed within the 3-month period weather the sinus rhythm was maintained. Clinical observation included 24-hour Holter monitoring performed on the next day, four weeks, and three months after the DC. In 7 days after DC, ambulatory ECG was performed. Patients reporting the symptoms suggesting the AF recurrence underwent additional resting ECG recording. As the confirmation of the arrhythmia recurrence, the resting ECG or Holter-ECG displaying the AF wave (including the paroxymal AF) was considered. The observation was completed after three months in patients who maintained the sinus rhythm or earlier in case of the recurrence of AF. The further treatment was recommended individually.

To develop the statistical analysis of the obtained data, STATISTICA 10.0 (StatSoft Inc., USA) was used. The compliance of the quantitative variables with normal distribution was checked using the Shapiro-Wilk test. To examine the relations between quality features, the *χ*^2^ test, *χ*^2^ test with Yates' correction, Fisher's exact test, or V2 test were performed. The variance homogeneity was proved using Levene's test. For the quantitative variable comparisons, Student's *t*-test and Mann–Whitney test were applied. Statistical significance assumed the results where the probability level (*p*) was lower than 0.05.

The study group consisted of patients undergoing DC in our center within the planned time period. The statistical power analysis was not performed because apelin is a parameter with not-normal distribution and nonparametric statistical tests were used.

## 3. Results and Discussion

### 3.1. Study Group Characteristics

The study group comprised 60 patients; it was divided depending on the effectiveness of cardioversion into SCD (successful DC), 45 (75%) patients, and NDC (nonsuccessful DC), 15 patients. Within the SCD group, the subgroups were distinguished depending on the time sinus rhythm maintenance after successful DC: up to 7 days (SDC-7); 7 to 30 days (SDC-30); over 90 days (SDC-90). The amount of each subgroup was SDC-7, 11 patients (18.34% of the total and 22.23% among patients in whom cardioversion was effective); SDC-30, 12 patients (20% of the total and 26.67% among patients in whom cardioversion was effective); SDC-90, 22 patients (36.67% of the total and 48.89% among patients in whom cardioversion was effective). These divisions were aimed at distinguishing patients in whom DC was effective in the long-term against the others.

Men predominated the study population accounting for 70%. The percentage of men in the various subgroups was within the range of 54.55% (SDC-7) to 80.00% (NDC). The youngest participant was 41 years and the oldest 86 years (mean 61.8 years).

The characteristics of the study population divided into subgroups is presented as shown in Table 1.

### 3.2. FA Duration

Atrial fibrillation duration in study population ranged from 1 to 24 months (mean 7.83). The longest average duration of arrhythmias was observed in the NDC group 9.93 (± 7.95) and the shortest 6.08 (± 6.07) in the SDC-30 group (see Table 1). In the study group, there was no relationship between the duration of arrhythmia and DC effectiveness (*p* = 0.590); moreover, it was found that the tested subgroups did not differ significantly concerning the FA duration.

Two thirds of respondents (66.67%) did not underwent electric cardioversion in the past. The majority (60%) of the remaining group had a DC only once. The maximum number of procedures was 9 in 1 patient. Comparing the numbers of patients, who underwent or not DC in the past within the study, only the subgroups SDC-7 (9.09% of patients had DC in the past) and SDC-90 (50% of patients had DC in the past) differed significantly (*p* = 0.0273).

There was no difference between the effectiveness of DC depending on the previous effective cardioversion (*p* = 0.5306). Moreover, there was no difference in apelin concentration depending on the previous DC (*p* = 0.8166).

### 3.3. Echocardiographic Parameters

It was examined whether there is a relationship between atrial dimensions assessed in echocardiography and the effectiveness of electrical cardioversion. The average values and median longitudinal and transverse dimensions of the left atrium (LA) and the right atrium (RA) are presented in Table 1.

Statistical analysis showed no significant difference in longitudinal dimension of the left or right atrium between any of the compared subgroups of patients differing in DC efficiency.

**Figure 1:**
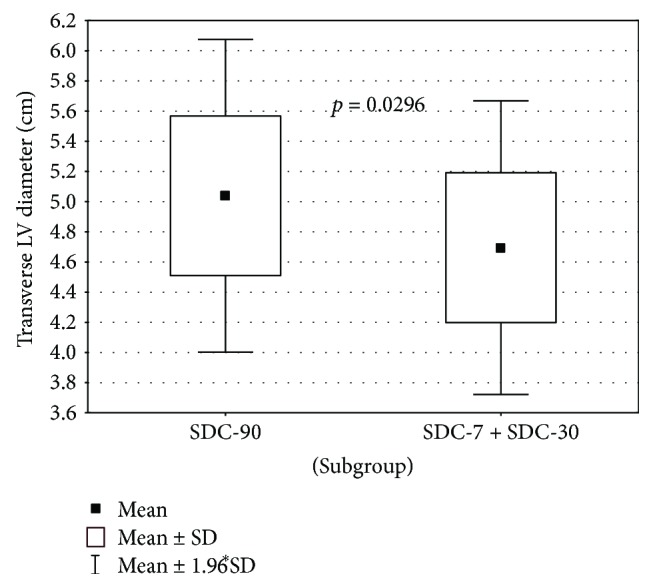
LA transverse diameter in subgroups SDC-90 versus SDC-7 + SDC-30.

**Figure 2:**
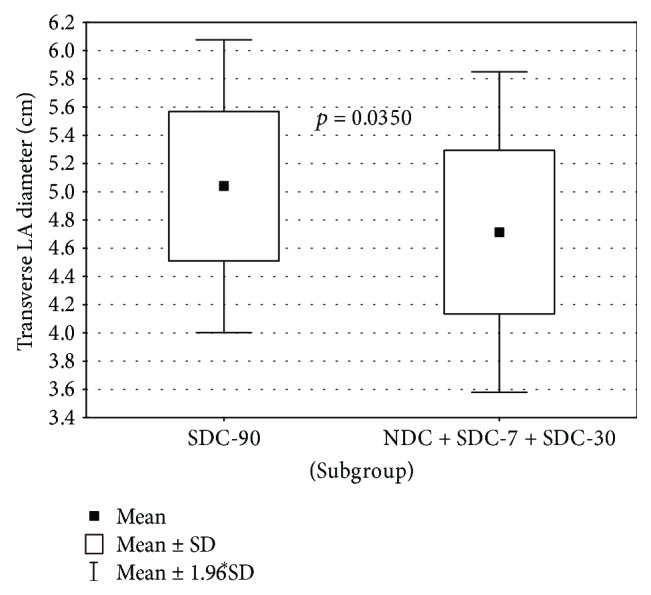
LA transverse diameter in subgroups SDC-90 versus NDC + SDC-7 + SDC-30.

Interestingly, it has been found that an SDC-7 + SDC-30 subgroup compared to the SDC-90 subgroup differs significantly concerning the value of the transverse dimension LA (*p* = 0.0296). Patients in the SDC-90 group had larger transverse LA dimension in comparison with SDC-7 + SDC-30 group. A statistically significant difference in LA transverse diameter was also found between subgroups NDC + SDC-7 + SDC-30 and SDC-90 (*p* = 0.0350). Here, consistently, the average size of LA was significantly higher in SDC-90 versus NDC + SDC-7 + SDC-30. These dependencies were illustrated in Figures 1 and 2.

It was also shown that the transverse RA dimension in SDC-30 group was significantly higher than in SDC-7 group (*p* = 0.0234). This stands also for the comparison between SDC-90 and SDC-7 groups, where the difference was close to statistical significance (*p* = 0.0578).

All the patients from the general study population had normal left ventricle function with ejection fraction of over 55%.

### 3.4. Comorbidity

Most of the observed patients were overweight or obese. BMI ranged from 23.74 to 44.98 (mean 30.70). Only 7 subjects had a normal body weight, 25 (41.67%) were overweight (BMI 25.0–30.0), and 28 were obese (BMI > 30.0), including two patients with morbid obesity (BMI > 40). The subgroups did not significantly differ concerning the body mass.

Eight patients (13.34%) had hyperthyroidism treatment currently or in the past, but at the moment of cardioversion, all were evaluated as euthyroid.

It was examined whether there is a correlation between the effectiveness of the DC and arterial hypertension occurrence in study population. Hypertension was diagnosed in 36 (60%) patients, with the greatest percentage of HA (66.67% and 63.84%) in the NDC and SDC-90 subgroup of SCD. There were no statistically significant correlation between the incidence of arterial hypertension and the effectiveness of treatment with DC.

Then the relation between DC efficiency and diabetes mellitus was subjected. Diabetes occurred in the total of 6 (10%) patients. A statistically significant difference in the incidence of diabetes among SDC-7 and SDC-90 (*p* = 0.0302) was observed. Diabetes was diagnosed in 27.27% in the SDC-7 subgroup but none of the patients from SDC-90 subgroup. The same proportion of people diagnosed with and without diabetes was observed for subgroups SDC-7 and SDC-30. This correlation, however, was not statistically significant (*p* = 0.0932).

Another coexisting disease entity was coronary artery disease, which appeared in a total of 5 (8.35%) patients, therein none of the patients in SDC-90 group. None of the patients had medical history of myocardial infarct. There were no statistically significant correlation between the incidence of coronary artery disease and the effectiveness of DC treatment in study population.

None of the patients had a medical history of the transient ischemic attack (TIA) or stroke.

The data on numbers and percentages of patients with known comorbidity in different study subgroups are presented in Table 1.

### 3.5. Apelin and proBNP Concentration in relation to DC Effectiveness

**Figure 3:**
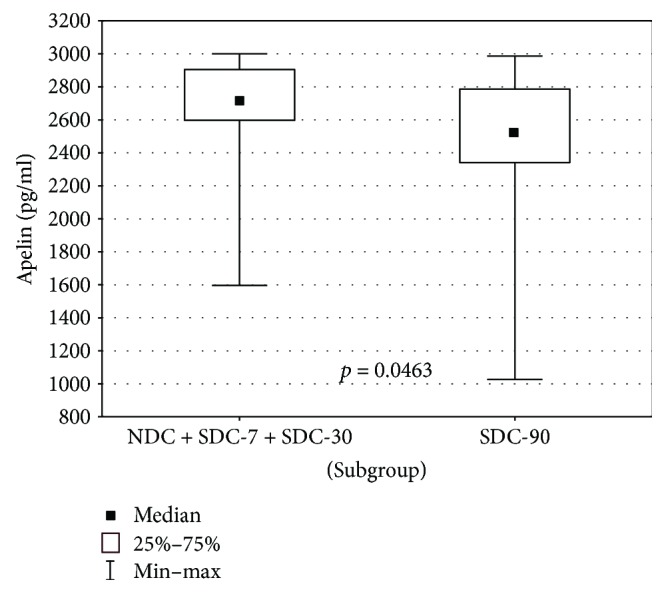
Apelin concentration in SDC-90 versus NDC + SDC-7 + SDC-30.

In a subsequent step of analysis, we sought the relationship between the apelin concentration and the effectiveness of electrical cardioversion. Mean and median values of plasma apelin in each group are shown in Table 2. It was found that the median value of apelin in the subset of patients from groups NDC + SDC-7 + SDC-30 is significantly higher than from group SDC-90 (*p* = 0.0463). This dependency was presented in Figure 3.

As to the concentration of apelin, proBNP was examined. Mean and median proBNP values in each group are shown in Table 2. Neither of the compared subgroup pairs revealed statistically significant correlation between the proBNP concentration and the DC effectiveness in our population. In Table 3, there were collected the *p* values for all conducted comparisons.

## 4. Discussion

The results of our study confirm the important role of apelin in the pathophysiology of cardiac arrhythmias. It was shown that the median, initial apelin level was significantly lower in patients who maintained sinus rhythm during the 3-month follow-up after the successful cardioversion, compared with patients in whom cardioversion was ineffective or who presented an arrhythmia recurrence. It has been shown that apelin affects the physiology of the circulatory system, including regulation of atrial cardiomyocytes action potential [13]. These reports are scarce, but they have become the base to search for apelin level variations in the abnormal heart rhythms. It has been documented that apelin plasma level is reduced in patients with isolated atrial fibrillation [14] and other supraventricular arrhythmias [16]. It is investigated whether apelin, among other biochemical markers, has its potential in predicting the risk of atrial fibrillation recurrence after electric cardioversion. So far, to our best knowledge, only one paper has been published on the subject. Falcone et al. [17] have achieved the opposite results, indicating an increased risk of AF recurrence in patients with lower baseline apelin.

Moreover, in our study group, there were no statistically significant differences in the values of proBNP depending on the DC efficiency, which is consistent with the results of some studies, for example, Shin et al. [6] and Tveit et al. [18]. However, some of the authors indicate the proBNP predictive value in predicting the FA recurrence [17, 19].

Discrepancies in the obtained results may result from the difference in the duration of AF, which was higher in patients from our study group. In addition, our study also included patients in whom DC was not effective. The variability in blood serum apelin concentration in patients with cardiac dysfunctions, including arrhythmia and heart failure, cannot be clearly predicted. It is due to the fact that apelin is involved both in pathophysiological and cardioprotective mechanisms. In heart failure, the level of apelin rises in the initial stage of the disease parallel with BNP and decreases in the advanced stage—those observations, in our opinion, might also be applied to patients with AF [11].

Despite the numerous data on the apelin multiple role in the physiology and pathophysiology of the cardiovascular system, from animal and human studies, the marker still remains poorly understood. The variability of its concentration in various pathophysiological conditions may be both a cause and a consequence of the phenomenon; therefore, their interpretation is not obvious.

There is a number of conditions, in which the apelin level variation has been proved including cardiovascular and metabolic disorders. It is difficult to separate these factors that undoubtedly affect the level of apelin in the group of cardiac patients.

At the same time, those conditions may also affect the course of atrial fibrillation, including the effectiveness of the cardioversion. Inability to eliminate all factors that may additionally affect the apelin level is undoubtedly the study limitation. However, we were able to reduce those effects as presented beneath.

Study groups did not differ significantly in terms of AF duration, and patients with a very large (>70 mm) atria dimensions were excluded. Examined patients did not differ significantly within the longitudinal atria dimensions, and their transverse atria dimensions were even larger in the subgroup with the sinus rhythm maintenance over 90 days.

Numerous studies demonstrated significantly higher plasma apelin level in obese patients [12, 20, 21]. Most of our study population was overweight or obese, but subgroups were not significant in between different concerning BMI.

In addition, apelin serum level was found to be decreased in human with primary hypertension [22, 23], coronary artery disease [24, 25], advanced heart failure [26], and increased in type 2 diabetes mellitus and impaired glucose tolerance subjects [27–29]. In our population, none of the patients had heart failure with symptoms above the first hemodynamic NYHA class, all had normal left ventricular systolic function in echocardiography. In addition, there was no statistical association between the arterial hypertension or coronary artery disease occurrence and the effectiveness of cardioversion; the largest proportion of HA patients occurred in the SDC-90 subgroup. Among comorbidities, only diabetes showed a statistically significant difference in the incidence between the SDC-7 and SDC-90 group and was more frequent in the SDC-7.

## 5. Conclusion

In conclusion, our data confirm the contribution of apelin to the atrial fibrillation pathophysiology. Conversely, compared to what has been previously documented, in our group initial, serum apelin level was significantly higher in patients who remained in the sinus rhythm over 90 days after the DC as compared to patients with the AF recurrence during follow-up and those in whom cardioversion was not effective. Furthermore, there were no differences in the concentration of proBNP depending on the DC efficiency.

A lower initial apelin serum concentration may indicate a group of patients with a more advanced stage of hemodynamic disorders caused by atrial fibrillation.

Basing on the currently available data, apelin cannot serve as an independent prognostic marker for the failure of the sinus rhythm maintenance strategy, but in the future, in addition to other biochemical and echocardiographic indices, it may be helpful in distinguishing a group of patients at risk of arrhythmia recurrence.

## Figures and Tables

**Table 1:** Study population characteristic.

	*N* (%)	Men (%)	FA duration (months); mean (±SD)	Previous DC (%)	Arterial hypertension *N* (%)	Diabetes mellitus *N* (%)	Coronary artery disease *N* (%)	Atrial dimensions							
								LA long. (cm)		LA trans. (cm)		RA long. (cm)		RA trans. (cm)	
								Mean (±SD)	Median (min–max)	Mean (±SD)	Median (min–max)	Mean (±SD)	Median (min–max)	Mean (±SD)	Median (min–max)
NDC	15 (25%)	12 (80%)	9.93 (±7.95)	4 (26.67%)	10 (66.67%)	3 (20%)	3 (20%)	6.3 (±0.38)	6.4 (5.80–6.58)	4.74 (±0.71)	4.7 (3.80–6.60)	5.81 (±0.45)	5.8 (5.20–6.70)	4.59 (±0.49)	4.4 (3.90–5.70)
SDC	45 (75%)	30 (66.67%)	7.13 (±5.95)	16 (35.56%)	26 (57.78%)	2 (4.4%)	2 (4.4%)	6.12 (±0.52)	6.2 (4.80–7.0)	4.86 (±0.54)	4.9 (3.30–6.10)	5.78 (±0.59)	5.9 (4.00–6.80)	4.52 (±0.71)	4.6 (3.00–5.65)
SDC-7	11 (18.32%)	6 (54.55%)	7.91 (±5.86)	1 (9.09%)	6 (54.55%)	3 (27.27%)	2 (18.18%)	6.05 (±0.62)	6 (4.80–7.0)	4.64 (±0.60)	4.8 (3.30–5.40)	5.92 (±0.36)	5.9 (5.40–6.50)	4.1 (±0.67)	4.1 (3.00–5.10)
SDC-30	12 (20%)	8 (66.67%)	6.08 (±6.07)	4 (33.33%)	6 (50%)	0	1 (8.33)	6.05 (±0.40)	6.2 (5.24–6.50)	4.75 (±0.40)	4.8 (3.94–5.50)	5.63 (±0.57)	5.7 (4.60–6.40)	4.74 (±0.59)	4.9 (3.50–5.50)
SDC-90	22 (36.67%)	16 (72.73%)	7.32 (±6.12)	11 (50%)	14 (63.64%)	0	0	6.19 (±0.54)	6.35 (5.24–7.0)	5.04 (±0.53)	5.11 (3.94–6.10)	5.79 (±0.68)	5.95 (4.00–6.80)	4.61 (±0.72)	4.7 (3.50–5.65)

**Table 2:** Apelin and proBNP serum concentration in study subgroups.

Subgroup	Apelin concentration (pg/ml)					proBNP concentration (pg/ml)				
	Mean	Median	Min.	Max.	SD	Mean	Median	Min.	Max.	SD
NDC	2736.61	2756	2243	2975	213.73	619.11	609.3	102	1284	373.24
SDC	2581.49	2662	1026	3000	373.86	733.28	636.3	70.78	2416	548.36
SDC-7	2672.64	2804	1597	3000	404.89	813.21	832.2	285.4	1711	395.91
SDC-30	2650.42	2664	2402	2974	146.03	526.98	412.3	102	1333	371.66
SDC-90	2490	2522	1026	2987	440.14	805.85	673.55	70.78	2416	670.72
SDC-7 + SDC-30	2661.04	2695	1597	3000	292.07	663.87	636.3	102	1711	402.1
NDC + SDC-7 + SDC-30	2688.33	2710.5	1597	3000	265.78	646.2	621.85	102	1711	386.44

**Table 3:** Apelin and proBNP serum level: *p* values for all conducted comparisons.

Compared subgroups	*p* value for apelin	*p* value for proBNP
NDC versus SDC	0.1252	0.6633
SDC-7 versus SDC-30	0.1858	0.0881
SDC-7 versus SDC-90	0.0793	0.3961
SDC-30 versus SDC-90	0.4027	0.3634
SDC-7 + SDC-30 versus SDC-90	0.1162	0.9547
**NDC + SDC-7 + SDC-30 versus SDC-90**	**0.0463**	0.8121

## Abbreviations

SDC: Successful cardioversion
NDC: Nonsuccessful cardioversion
SDC-7: Sinus rhythm maintenance within 7 days
SDC-30: Sinus rhythm maintenance 7 to 30 days
SDC-90: Sinus rhythm maintenance over 90 days.

## References

[1] Camm A. J., Lip G. Y., De Caterina R. (2012). 2012 focused update of the ESC Guidelines for the management of atrial fibrillation: an update of the 2010 ESC Guidelines for the management of atrial fibrillation. Developed with the special contribution of the European Heart Rhythm Association. *European Heart Journal*.

[2] Go A. S., Hylek E. M., Phillips K. A. (2001). Prevalence of diagnosed atrial fibrillation in adults: national implications for rhythm management and stroke prevention: the anticoagulation and risk factors in atrial fibrillation (ATRIA) study. *JAMA*.

[3] Stewart S., Hart C. L., Hole D. J., McMurray J. J. V. (2002). A population-based study of the longterm risks associated with atrial fibrillation: 20-year follow-up of the Renfrew/Paisley study. *The American Journal of Medicine*.

[4] Gilewski W., Sinkiewicz W. (2013). The electrical cardioversion of atrial fibrillation after 50 years since initial application: contemporary knowledge. *Kardiologia Polska*.

[5] Hijazi Z., Oldgren J., Siegbahn A., Granger C. B., Wallentin L. (2013). Biomarkers in atrial fibrillation: a clinical review. *European Heart Journal*.

[6] Shin D. I., Jaekel K., Schley P. (2005). Plasma levels of NT-pro-BNP in patients with atrial fibrillation before and after electrical cardioversion. *Zeitschrift für Kardiologie*.

[7] Zografos T., Katritsis D. (2013). Natriuretic peptides as predictors of atrial fibrillation recurrences following electrical cardioversion. *Arrhythmia & Electrophysiology Review*.

[8] Tatemoto K., Hosoya M., Habata Y. (1998). Isolation and characterization of a novel endogenous peptide ligand for the human APJ receptor. *Biochemical and Biophysical Research Communications*.

[9] Kleinz M. J., Davenport A. P. (2005). Emerging roles of apelin in biology and medicine. *Pharmacology & Therapeutics*.

[10] O’Carroll A. M., Lolait S. J., Harris L. E., Pope G. R. (2013). The apelin receptor APJ: journey from an orphan to a multifaceted regulator of homeostasis. *The Journal of Endocrinology*.

[11] Chen M., Ashley E., Deng D. (2003). Novel role for the potent endogenous inotrope apelin in human cardiac dysfunction. *Circulation*.

[12] Boucher J., Masri B., Daviaud D. (2005). Apelin, a newly identified adipokine up-regulated by insulin and obesity. *Endocrinology*.

[13] Cheng C. C., Weerateerangkul P., Lu Y. Y. (2013). Apelin regulates the electrophysiological characteristics of atrial myocytes. *European Journal of Clinical Investigation*.

[14] Ellinor P. T., Low A. F., Macrae C. A. (2006). Reduced apelin levels in lone atrial fibrillation. *European Heart Journal*.

[15] Gurger M., Celik A., Balin M. (2014). The association between apelin-12 levels and paroxysmal supraventricular tachycardia. *Journal of Cardiovascular Medicine*.

[16] Kallergis E. M., Manios E. G., Kanoupakis E. M. (2010). Effect of sinus rhythm restoration after electrical cardioversion on apelin and brain natriuretic peptide prohormone levels in patients with persistent atrial fibrillation. *The American Journal of Cardiology*.

[17] Falcone C., Buzzi M. P., D’Angelo A. (2010). Apelin plasma levels predict arrhythmia recurrence in patients with persistent atrial fibrillation. *International Journal of Immunopathology and Pharmacology*.

[18] Tveit A., Seljeflot I., Grundvold I., Abdelnoor M., Arnesen H., Smith P. (2009). Candesartan, NT-proBNP and recurrence of atrial fibrillation after electrical cardioversion. *International Journal of Cardiology*.

[19] Sanna T., Sonaglioni A., Pieroni M. (2009). Baseline NT-pro-BNP levels and arrhythmia recurrence in outpatients undergoing elective cardioversion of persistent atrial fibrillation: a survival analysis. *Indian Pacing and Electrophysiology Journal*.

[20] Castan-Laurell I., Dray C., Attané C., Duparc T., Knauf C., Valet P. (2011). Apelin, diabetes, and obesity. *Endocrine*.

[21] Castan-Laurell I., Boucher J., Dray C., Daviaud D., Guigné C., Valet P. (2005). Apelin, a novel adipokine over-produced in obesity: friend or foe?. *Molecular and Cellular Endocrinology*.

[22] Przewlocka-Kosmala M., Kotwica T., Mysiak A., Kosmala W. (2011). Reduced circulating apelin in essential hypertension and its association with cardiac dysfunction. *Journal of Hypertension*.

[23] Papadopoulos D. P., Mourouzis I., Faselis C. (2013). Masked hypertension and atherogenesis: the impact of apelin and relaxin plasma levels. *Journal of Clinical Hypertension*.

[24] Kadoglou N. P. E., Lampropoulos S., Kapelouzou A. (2010). Serum levels of apelin and ghrelin in patients with acute coronary syndromes and established coronary artery disease—KOZANI STUDY. *Translational Research*.

[25] Li Z., Bai Y., Hu J. (2008). Reduced apelin levels in stable angina. *Internal Medicine*.

[26] Chong K. S., Gardner R. S., Morton J. J., Ashley E. A., McDonagh T. A. (2006). Plasma concentrations of the novel peptide apelin are decreased in patients with chronic heart failure. *European Journal of Heart Failure*.

[27] Telejko B., Kuzmicki M., Wawrusiewicz-Kurylonek N. (2010). Plasma apelin levels and apelin/APJ mRNA expression in patients with gestational diabetes mellitus. *Diabetes Research and Clinical Practice*.

[28] Ma W. Y., Yu T. Y., Wei J. N. (2014). Plasma apelin: a novel biomarker for predicting diabetes. *Clinica Chimica Acta*.

[29] Kadoglou N. P. E., Tsanikidis H., Kapelouzou A. (2010). Effects of rosiglitazone and metformin treatment on apelin, visfatin, and ghrelin levels in patients with type 2 diabetes mellitus. *Metabolism*.

